# Individual differences in social information gathering revealed through Bayesian hierarchical models

**DOI:** 10.3389/fnins.2013.00165

**Published:** 2013-09-12

**Authors:** John M. Pearson, Karli K. Watson, Jeffrey T. Klein, R. Becket Ebitz, Michael L. Platt

**Affiliations:** ^1^Department of Neurobiology, Center for Cognitive Neuroscience, and Duke Institute for Brain Sciences, Duke UniversityDurham, NC, USA; ^2^Bowles Center for Alcohol Studies, University of North Carolina at Chapel Hill School of MedicineChapel Hill, NC, USA; ^3^Department of Biological Anthropology, Duke UniversityDurham, NC, USA

**Keywords:** Bayesian hierarchical model, pay-per-view, social information

## Abstract

As studies of the neural circuits underlying choice expand to include more complicated behaviors, analysis of behaviors elicited in laboratory paradigms has grown increasingly difficult. Social behaviors present a particular challenge, since inter- and intra-individual variation are expected to play key roles. However, due to limitations on data collection, studies must often choose between pooling data across all subjects or using individual subjects' data in isolation. Hierarchical models mediate between these two extremes by modeling individual subjects as drawn from a population distribution, allowing the population at large to serve as prior information about individuals' behavior. Here, we apply this method to data collected across multiple experimental sessions from a set of rhesus macaques performing a social information valuation task. We show that, while the values of social images vary markedly between individuals and between experimental sessions for the same individual, individuals also differentially value particular categories of social images. Furthermore, we demonstrate covariance between values for image categories within individuals and find evidence suggesting that magnitudes of stimulus values tend to diminish over time.

## 1. Introduction

Over the last decade, neuroscientists have made increasing use of model-based analysis methods to capture the dynamics of neural signals, particularly in studies of choice (Schultz et al., 1997; Montague et al., 2004; Daw et al., 2006; Kennerley et al., 2006; Behrens et al., 2007; Quilodran et al., 2008; Krajbich et al., 2009; Pearson et al., 2009). Typically, parameters derived from models fitted to subjects' behavior are used as regressors in models of neural dynamics, and studies test the hypothesis that these inferred parameters are encoded in experimental measures such as neuronal firing rates, EEG, or the BOLD signal (Friston et al., 2003). However, choice behavior in both humans and non-human animals has proven notoriously variable within and between experimental sessions, resulting in highly variable estimates of subjects' individual model parameters. Perhaps just as importantly, the correctness of correlations between neural measures and model-derived parameters depends crucially on obtaining accurate and robust estimates of the latter. Overfitted models are likely to produce inaccurate and fragile parameter estimates, resulting in overstated or spurious correlations, and to generalize poorly to unseen data, inflating significance at the cost of robustness.

At the same time, studies of individual differences in behavior, spurred by advances in genomics, have become a topic of increasing interest in neuroscience (Hariri et al., 2002; Buckholtz et al., 2007; Hariri, 2009). Yet constraints in data collection have limited the ability of researchers to draw statistically robust conclusions, particularly in experimental designs where the amount of data per subject is necessarily large, for instance, when a behavioral model must be fit to each subject's data.

In between-subjects designs, the solution to these dilemmas has been to treat subject identity as a random effect in so-called “mixed effects models,” in which population variation across variables of interest is modeled explicitly (Pinheiro and Bates, 2000). However, these techniques are only rarely applied in animal studies, where the unit of analysis is the single neuron or single experimental session, and somewhat more frequently in human studies when more sophisticated subject-specific models must be fit (Friston et al., 2005). As a result, experimenters most often pool all data across a single individual to estimate parameters such as risk aversion and discount rates (Deaner et al., 2005; Klein et al., 2008; Louie and Glimcher, 2010; Pearson et al., 2010; Watson and Platt, 2012; Klein and Platt, 2013), ignoring variation across sessions.

Yet sophisticated techniques exist that correctly account for these effects, allowing for accurate estimation of correlated variation across both subjects and sessions. One such technique, Bayesian Hierarchical Modeling, is used widely in the social sciences to account for variance both within and between individuals (Gelman et al., 2003; Gelman and Hill, 2007), and multiple software packages allow for easy specification of models (Plummer, 2003; Shiffrin et al., 2008; Lunn et al., 2012). Such models incorporate three key advantages for neuroscientists wishing to accurately account for sources of variation in behavioral data: First, they correctly capture the covariance structure of the task. Experimental sessions performed by the same individual are neither independent nor identical, requiring some accounting for repeated measures effects. Second, such models allow for subject- or session-specific estimates of key parameters, even when session data may be incomplete or missing. In other words, statements about individual differences become feasible with fewer data. Third, these models optimally (in a Bayesian sense) interpolate between no pooling (treating each session, say, as independent) and complete pooling (treating all sessions as identical), allowing “prior” information from other sessions to be used in fitting a given day's data. In practice, as we show below, this allows us to recover reasonable parameter estimates for days with noisy and unruly data, sessions that might previously have been excluded from analysis.

Here, we take as a case study choice data from a laboratory task performed for multiple sessions in multiple rhesus macaques. In previous studies (Deaner et al., 2005; Klein et al., 2008; Watson and Platt, 2012; Klein and Platt, 2013), we have used estimates of value derived from this behavior as potential correlates of single-unit neural activity, treating values within each session as independent. In practice, this means that days with atypical behavior result in poor model fits and unrealistic value estimates. In the following, we show that hierarchical models allow us not only to make valuable statements about individual differences in choice behavior, but to tame ill-behaved fits via partial pooling, leading to better-behaved models and more reliable characterizations of behavior. Such techniques hold promise not only for theoretical investigations of behavior, but for more systematic and principled studies of differences between individuals.

## 2. Materials and methods

### 2.1. Behavioral task

We combined choice data from 206 sessions of a laboratory-based social valuation task performed by *N* = 8 male rhesus macaques housed at Duke University (subjects E, Os, Ot, D, S, C, B, and N; *N* = 60, 32, 51, 23, 14, 10, 8, and 8 sessions, respectively). Details of the behavioral paradigm have been published elsewhere (Deaner et al., 2005; Klein et al., 2008; Watson and Platt, 2012; Klein and Platt, 2013), but briefly, subjects made repeated decisions between options resulting in either juice alone or juice plus the opportunity to view a social image. The difference in juice amounts between the two options was systematically varied in a block design, along with the content of the social images. In each block, social images were drawn from pools corresponding to four image categories: dominant males, subordinate males, female perinea, and a gray square (control). For our analysis, we used aggregated choice counts in each session, tabulated for each unique combination of juice difference and image category.

For behavioral analysis, we are interested in the indifference point or point of subjective equality (PSE), at which subjects choose the image plus juice and juice only options at equal rates. To examine this quantitatively, we define the juice differential *dv* as

(1) $$dv\equiv {\text{juice}}_{\text{image}}-{\text{juice}}_{\text{blank}},$$

so that positive *dv* implies a higher juice amount for the juice plus image option. Indifference occurs when *dv* is equal in magnitude but opposite in sign to the image value *v*. Thus we measure image value in units of foregone juice, which in our experiment was controlled by the open time of a solenoid allowing for a roughly constant rate of juice delivery. As a result, we report image values in equivalent milliseconds of juice access. However, for computational purposes (and in the equations below), we use seconds of juice access as our measurement scale.

### 2.2. Hierarchical model

We fit monkeys' choice behavior with a logistic regression model that included mean image values specific to each monkey and category (*V*_*mc*_), values specific to each session (*v*_msc_), session-to-session variability for each monkey (σ^2^_*m*_), and monkey-specific overdispersion in choice variance (ω^2^_*m*_). Each session consisted of a total of *N*_*c*_ choices for each social image category, of which *n*_*c*_ were for the image plus juice option. Thus, for a particular trial set involving image category *c* in session *s* for monkey *m*:

(2) $$v_{\text{msc}}\sim N\left(V_{mc},\sigma_{m}^{2}\right)$$

(3) $$\eta_{\text{msc}}=\frac{dv+v_{\text{msc}}}{\tau_{s}}$$

(4) $$\text{logit }p_{\text{msc}}\sim N\left(\eta_{\text{msc}},\omega_{m}^{2}\right)$$

(5) $$n_{\text{msc}}\sim \text{binomial}\left(p_{\text{msc}},N_{\text{msc}}\right)$$

That is, image values for each image category for each monkey each day are drawn from a normal distribution with mean specific to the monkey (*V*_*mc*_) and category and variation specific to the monkey (σ^2^_*m*_) (2). These image values are then combined with the juice differential (*dv*) and scaled by a session-specific normalization (τ_*s*_) to produce a choice utility (3). (The distribution of this scaling parameter is assumed to be the same for all monkeys. Thus, τ_*s*_ carries no *m* index.) The probability of choosing the image plus juice option is then related to this utility by added variance (ω^2^_*m*_, specific to each monkey) (4). This superadded variance captures variability of the choice behavior over and above what would be predicted from the binomial distribution (5).

### 2.3. Time variation

To allow for the possibility that stimuli lost their potency across sessions, we allowed for an explicit time dependence in mean image value:

(6) $$v_{\text{msc}}\sim N\left(V_{mc}+\alpha_{mc}t,\sigma_{m}^{2}\right)\text{​},$$

where *t* indexes the date of each session for each subject. That is, a slope parameter was added for each monkey and category (for a total of 32 parameter across our subject pool). To speed convergence of the algorithms, these dates were rank ordered and z-scored, implying that *t* has mean 0 and unit variance (though we report values in units of ms juice per session). We specified priors on the time rate of change in image value as

(7) $$\alpha_{mc}\sim N(0,0.01)\text{​}.$$

Finally, we examined correlations among session-to-session image values across categories. Because Bayesian estimates of correlations are often slow to converge (Gelman and Hill, 2007), we performed an exploratory analysis for each subject as follows: For each subject, we created paired scatterplots of image category values by sampling from their joint posterior distribution. That is, each scatterplot point represents a draw from the joint distribution of the two image values *P*(*v_i_*, *v_j_*), where *i* and *j* are image categories. More specifically, we plotted 1000 samples drawn from the combined set of samples across all sessions, {**v** = (*v*_1_, *v*_2_, …, *v_c_*)|**v** ~ *P_s_*(**v**) for some session *s*}.

### 2.4. Priors

Furthermore, our model is a Bayesian model that requires us to specify prior distributions for each model parameter. For purposes of simulation, image values were measured in seconds of juice access. In specifying these priors, we have attempted to make only minimal assumptions regarding plausible ranges of prior parameters. These assumptions are based on previous reports of the same data, where image values ranged from near 0 to tens of ms, but represent much weaker restrictions. In most cases, the distributions chosen vary only minimally over a large range of potential parameter values, resulting in estimates that are overwhelmingly determined by data, not prior parameters or shapes.

Priors for our simulations were chosen as follows:

(8) $$V_{mc}\sim N(0,0.01)$$

(9) $$\sigma_{m}\sim U(10^{-6},1)$$

(10) $$\log\omega_{m}\sim U(-6,2)$$

(11) $$\tau_{s}\sim t_{+}(\ell ,\varsigma ,\nu )$$

(12) ℓ~U(0,0.5)

(13) $$\varsigma \sim U(10^{-4},0.1)$$

(14) ν~U(0.1,50)​,

where *U* is the uniform distribution, *t*_+_(ℓ, ς, ν) is the positively-truncated *t* distribution with location, scale, and degrees of freedom ℓ, ς, and ν, respectively.

That is, we have specified a variance for the prior on image values *V* that is σ^2^ = 0.01 *s*^2^, equivalent to a standard deviation of 100 ms, far larger than the largest *dv*. Likewise, the standard deviation across sessions, σ_*m*_ is only assumed to lie somewhere in the interval between 10^−3^ and 1000 ms. Similarly, the overdispersion in normalized decision utilities, ω_*m*_, is assumed uniformly distributed on a logarithmic scale spanning a large range of values. Finally, in choosing a prior distribution on utility normalizations, τ_*s*_, corresponding to the widths of choice curves, preliminary analyses with conventional fits suggested a more outlier-heavy distribution than the typical normal or gamma forms. Following a suggestion in Gelman and Hill (2007), we thus modeled these as a truncated *t* distribution, which more accurately captures the presence of outliers, with the potential of approaching a truncated normal distribution as the degrees of freedom, ν, grow large. In addition, we have allowed this distribution to be peaked away from zero by including a location parameter ℓ. Naturally, the restriction that τ_*s*_ be positive requires that we truncate and normalize the distribution to the positive real line.

What is most important to note is that none of the parameter estimates produced by the model depends sensitively on the particular priors used, so long as these distributions do not constitute a strong restriction on the data. In our case, we have chosen only weakly informative priors based on the ranges of parameters observed in prior studies. In all cases, these priors permit much larger ranges of *a priori* variation than seen in that work.

### 2.5. Sampling: theory

Computational approaches to the problem of Bayesian estimation are numerous and discussed in many introductory texts (Chib and Greenberg, 1995; Gelman et al., 2003; MacKay, 2003). Here, we focus on the basic idea behind the most successful of these approaches, Markov Chain Monte Carlo (MCMC).

In Bayesian inference, the problem is that of sampling from a distribution *p*(**x**) when that distribution is too complicated to calculate in closed form. Clearly, if it is possible to sample from such a distribution, it is possible to estimate its shape by taking many samples (though this may be intractable for distributions over very high-dimensional spaces). The key insight responsible for MCMC is that this sampling can be implemented by clever use of an entity called the Markov Chain. A Markov Chain is a set of random variables {**x**_*t*_} where the dependence of a given point in the sequence on its past, *p* (**x_t + 1_**|**x_t_, x_t − 1_**, …) takes on a particularly simple form:

(15) $$p\left(x_{t+1}|x_{t},x_{t-1},\ldots \right)=p\left(x_{t+1}|x_{t}\right)\text{​}.$$

That is, the value of the chain at **x_t_** depends only on the value of the chain immediately previous, and the entire process is characterized by a matrix of transition probabilities. Just as importantly, for a large class of Markov chains, as *t* grows large, the distribution over samples settles down to a stable form, π(**x**) (the one left invariant by the transition matrix). That is to say, the sequence of elements from the Markov chain constitute an unbiased sample from this stable distribution, even though sequential samples are correlated.

As Chib and Greenberg (1995) note, the insight of MCMC was to turn this situation on its head. Rather than ask what form π(**x**) takes, given the Markov chain, we can try to find a Markov chain such that π(**x**) = *p*(**x**). If we have such a chain, we can sample the distribution of interest by simply applying the transition matrix. In the simplest form of the MCMC algorithm, Gibbs sampling, this is accomplished by sequentially drawing each individual parameter in the vector **x** from its distribution conditioned on the other parameters:

(16) $$x_{i}\prime \sim p\left(x_{i}|x_{-i}\right)$$

where *x*_*i*_ is the *i*th parameter of the distribution and *x*_− *i*_ is the remaining set of parameters. In other words, valid samples can be drawn by altering individual parameters one by one, each time drawing from a univariate probability distribution while holding the values of all other variables fixed. It can then be shown that the resulting vectors, derived by changing one element at a time, constitute a sample from the desired distribution *p*(**x**).

### 2.6. Sampling: details

For each parameter of interest in our model, we calculated posterior distributions by drawing samples using Gibbs sampling (Gelman et al., 2003; Gelman and Hill, 2007). Specifically, we used the R interface (rjags) to the JAGS (Just Another Gibbs Sampler) sampling package (Plummer, 2003). For each posterior distribution of interest, we collected 5000 samples from five chains by running each chain for 20,000 samples with a thinning fraction of 20. Sampling algorithms were adapted for 1000 samples (JAGS employs multiple efficiency-improving tweaks to standard Gibbs, such as block sampling, that require an initial adaptation phase), followed by a burn-in of 10,000 samples, which were discarded. We monitored convergence both by ratios of within-chain and between chain variance, as captured in the effective sample size and $\hat{R}$ statistics (Gelman et al., 2003; Gelman and Hill, 2007). All chains for variables of interest had $\hat{R}$ < 1.1, indicating that sampling had converged.

In addition, for posterior predictive checking, we simulated 500 fictitious sessions from our model (5000 samples, 5 chains, thinned by a factor of 10). That is, we drew 2500 samples each from (11), (4), and (2) for each monkey and each image category. This allowed us to compare choice curves produced by monkeys in our real data set with curves predicted by our generative model, allowing us to ask whether our observed data were typical for sessions generated from the final model. These simulated data were then fit according to a standard logit choice model

(17) $$\text{logit }p\sim \beta_{0}+\beta_{1}dv$$

to produce choice curves as a function of value difference between the two options.

### 2.7. Model comparison and fit

For each level in our model containing variance (choice counts, scaled utilities, across sessions), we calculated *R*^2^ and pooling fractions λ. For a given quantity (*x* = *n*, η, *v*):

(18) $$R^{2}=1-\frac{\text{var}(\epsilon_{x})}{\text{var}(x)}$$

(19) $$\lambda =1-\frac{\text{var}(E_{u}[\epsilon_{x}])}{\text{var}(\epsilon_{x})}\text{​},$$

where ϵ_*x*_ is the model residual for *x* and *E*_*u*_[·] is the expectation within units (counts, utilities, sessions). That is, *R*^2^ is one minus the ratio of residual variance to total variance, and λ is one minus the ratio of the between-units residual variance to the total residual variance. Like *R*^2^, λ ranges between 0 and 1, where λ = 1 indicates complete pooling (all units treated identically) and λ = 0 indicates no pooling (all units treated independently) (Gelman and Hill, 2007). That is, λ captures the extent to which individual units are pooled toward the group mean.

The problem of comparisons between our hierarchical Bayesian model and standard regression approaches is a difficult one. Because our model (and its variants) nominally contain many more parameters than standard no-pooling models (one choice curve per session), assessments of model performance must not only penalize for complexity but correctly estimate the number of effective degrees of freedom. Note that for hierarchical models such as ours, this may mean fewer “effective” degrees of freedom than model parameters, since the hierarchical distribution assumptions mean that these parameters are far from independent.

Thus, to compare between models, we use the Deviance Information Criterion (DIC), available in JAGS and proposed as a generalization of criteria like AIC and BIC more appropriate for hierarchical models (Gelman et al., 2003; Berg et al., 2004). Like AIC and BIC, DIC can be viewed as a penalized log likelihood, trading off model fit against model complexity. Also, like AIC and BIC, lower numbers indicate better “fits,” meaning more accurate generalization of a model to unseen data.

To perform our model comparisons, we again used Gibbs sampling (dic.samples command in rjags: 5000 iterations, thinning fraction of 10, 5 chains, for a total of 2500 samples) following both rounds of sampling above to estimate the DIC for variants of our model. These variants included our main model as described above, the model including time trend in image value, a model ignoring subject as a factor, a model ignoring category as a factor, and a model ignoring both subject and category as factors (i.e., pooling only across sessions, without regard to image type or monkey). Finally, in order to provide some comparison with conventional methods, we also estimated DIC for a model with no pooling across any variable (each session fit independently), a model collapsed across category (fit separately for each monkey), and a model collapsed across monkey (fit separately for each category). These were Bayesian models that fit subsets of the data separately, with no hierarchy. As such, it required specifying priors on the relevant parameters, which tend to regularize fits and so reduce overfitting more than conventional methods. Collectively, the DIC values returned from these simulations allowed us to assess the relative effects on predictive power of including more complexity in our model.

### 2.8. Data and code

The combined data, along with code used to produce model fits and figures for the paper, are publicly available: http://www.github.com/jmxpearson/ppv.

## 3. Results

**Figure 1:**
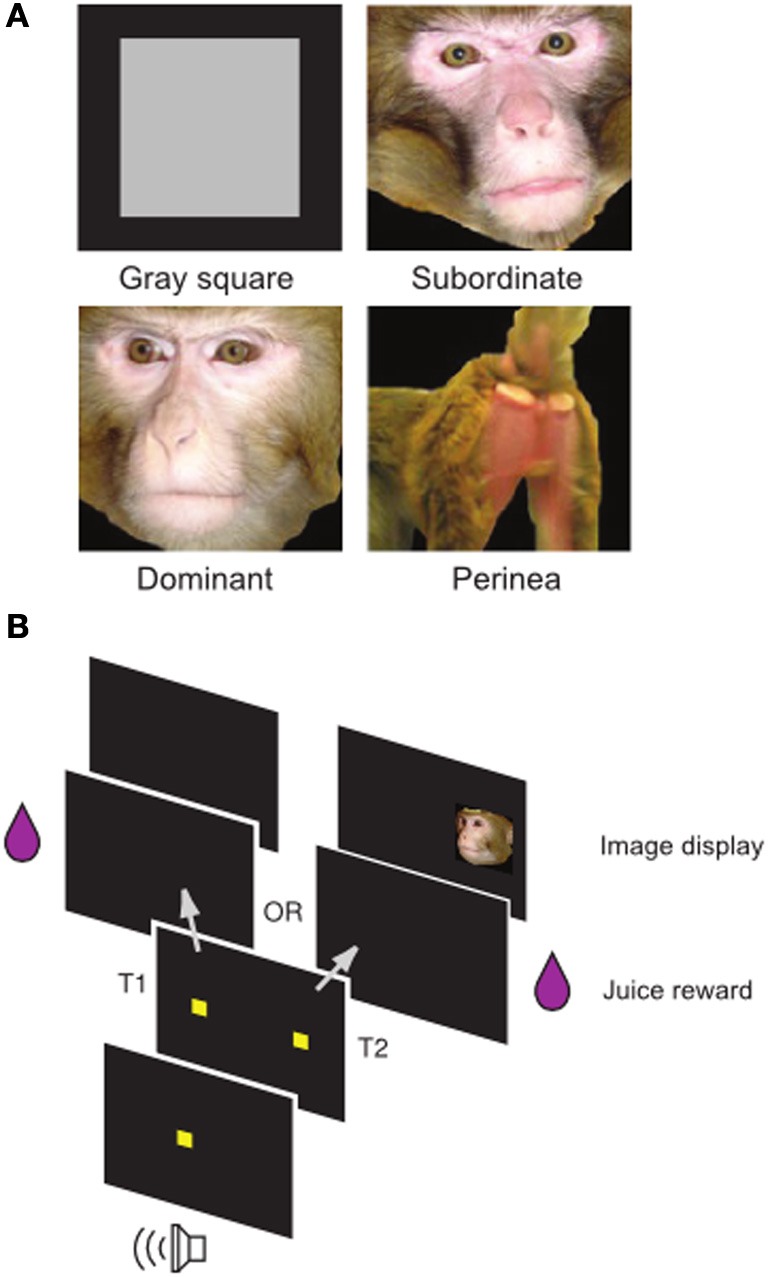
**Social Value Task. (A)** Examples of non-social control (gray square) and social images (subordinate male, dominant male, female perinea) used in the task. **(B)** Temporal structure of the task. Following a fixation cue, subjects made an eye movement to one of two targets. Selection of target T1 resulted in juice alone, while selection of target T2 resulted in juice plus an image. Adapted from Watson and Platt (2012).

Using published (Klein et al., 2008; Watson and Platt, 2012; Klein and Platt, 2013) and unpublished data from a well-known social image valuation task in rhesus macaques (Figure 1), we fit a Bayesian Hierarchical Model to choice data to estimate single-session image values in each animal. Briefly, subjects repeatedly chose between two options, a visual target resulting in juice delivery and a visual target resulting in juice delivery plus the display of a social image from one of four categories (Neutral, Female, Dominant Male, Subordinate Male). Differences in juice amounts for the two options varied systematically in a blocked design.

**Figure 2:**
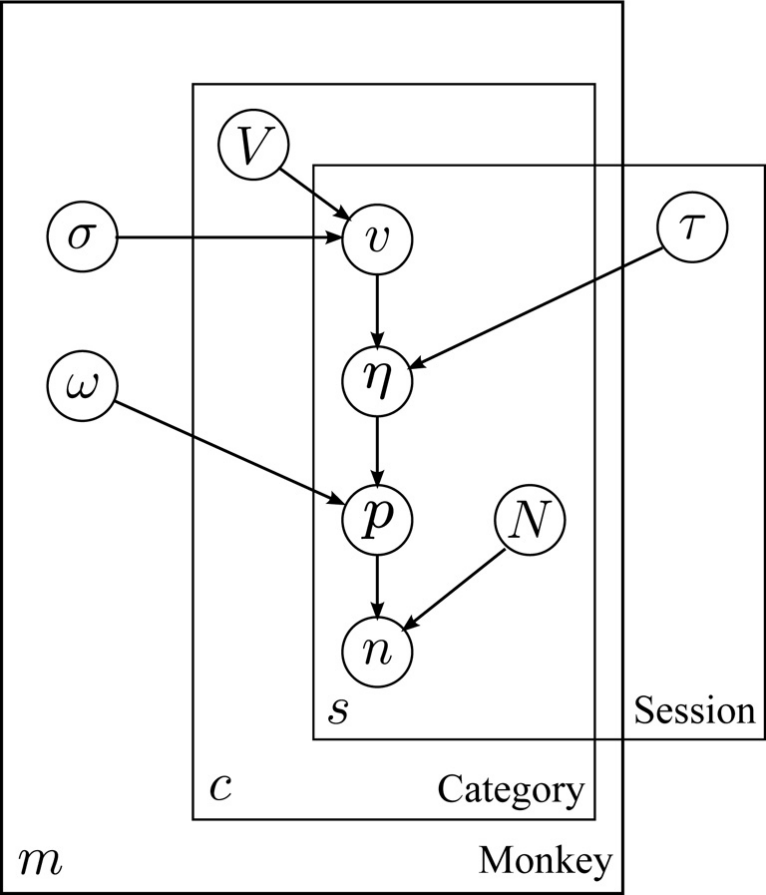
**Structure of the hierarchical model**. Plate model diagram of the hierarchical model for behavior. Circles represent model parameters. Arrows represent model dependencies. Plates enclose variables indexed by monkey, session, and image category (*m*, *s*, and *c*, respectively). Abbreviations: *V*: mean image value, σ: standard deviation of image value across sessions, *v*: session image value, *eta*: utility, τ: utility scaling factor, *p* image option choice probability, ω: choice overdispersion, *n*: number of image option choices, *N*: total number of choices.

Figure 2 depicts the relationship between variables in the model. Image values (*v*) for each day are assumed to be drawn from normal distributions specific to each subject and category. Choice variability, in the form of a logistic curve width (τ), is likewise assumed to vary between sessions in a manner common to all subjects. Choice probabilities are assumed to be given by an overdispersed logit model (ω), based on the difference in subjective value (juice plus image equivalent value, η) between two options.

Table 1 presents level-by-level summary statistics for the model fit. The model captures nearly all variance (*R*^2^) at the level of individual counts, largely due to trial-by-trial variations in value fit by the model (see Methods). But it also captures large percentages of the category (utility) and session-to-session variance as well. Moreover, λ values for each of the three levels show that pooling is strongest at the level of count data (strongly pooled toward the aggregate of all choices) and at the session level, indicating that information from all sessions for a given subject was crucial in fitting day-to-day estimates of image value.

**Table 1:** **Model fitting metrics for each level of the hierarchical model (session-to-session variance, variance in the scaling of utility, and binomial variance in choices, respectively)**.

**Level**	**Variance explained (*R*^2^)**	**Pooling (λ)**
Session	0.42	0.87
Utility	0.75	0.18
Counts	0.97	0.93

Variance explained at each level is the ratio of model variance to actual variance in the data at each level. Pooling is a measure of how much the data at each level are pooled toward the group mean (pooling = 1) or fit independently (pooling = 0).

**Figure 3:**
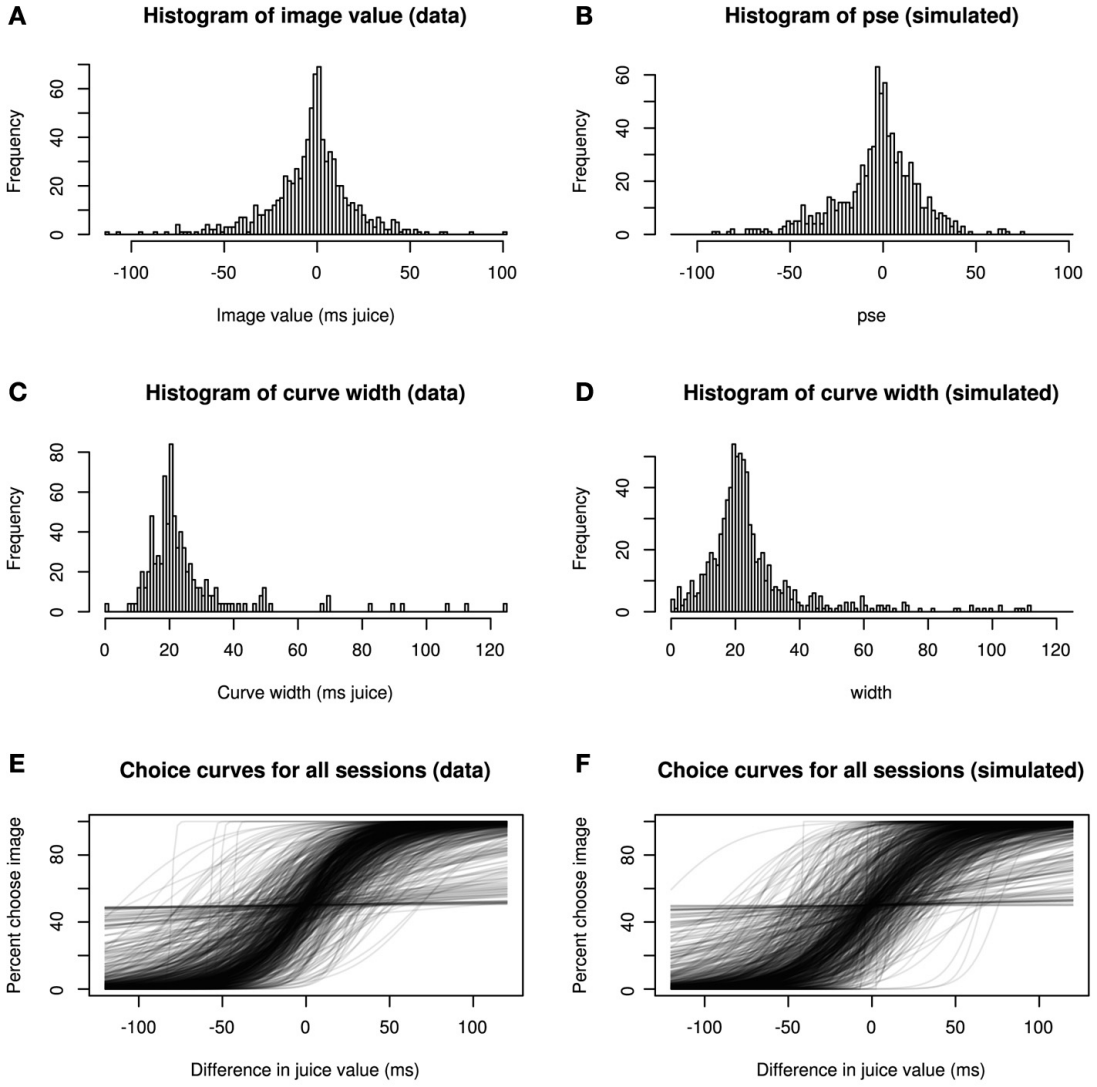
**Hierarchical model recapitulates choice data**. Three statistical characterizations of choice behavior based on observed data **(A,C,E)** and posterior predictive data simulated from the model **(B,D,F)**. In **(A,B)**, image values, collapsed across category, are plotted for real and simulated data. In **(C,D)**, histograms depict, widths (inverse precision) for choice curves fitted to the data. Finally, **(E,F)** show choice curves fit to both real and simulated data using standard logistic methods. In each case, the data produced by the generative model capture key features of the real data.

Figure 3 confirms this goodness-of-fit by a series of posterior predictive checks. Posterior predictive checking, rather than asking how well a model fits a given data set, asks how typical the observed data are for the output of the fitted model (Gelman and Hill, 2007). In other words, we compared descriptive statistics from our model fit with those of simulated data generated *de novo* from the final model. Clearly, the distributions of image values, choice curve widths, and the shapes of the choice curves themselves show strong consistency, indicating that our model accurately captures major sources of variation in the data.

**Figure 4:**
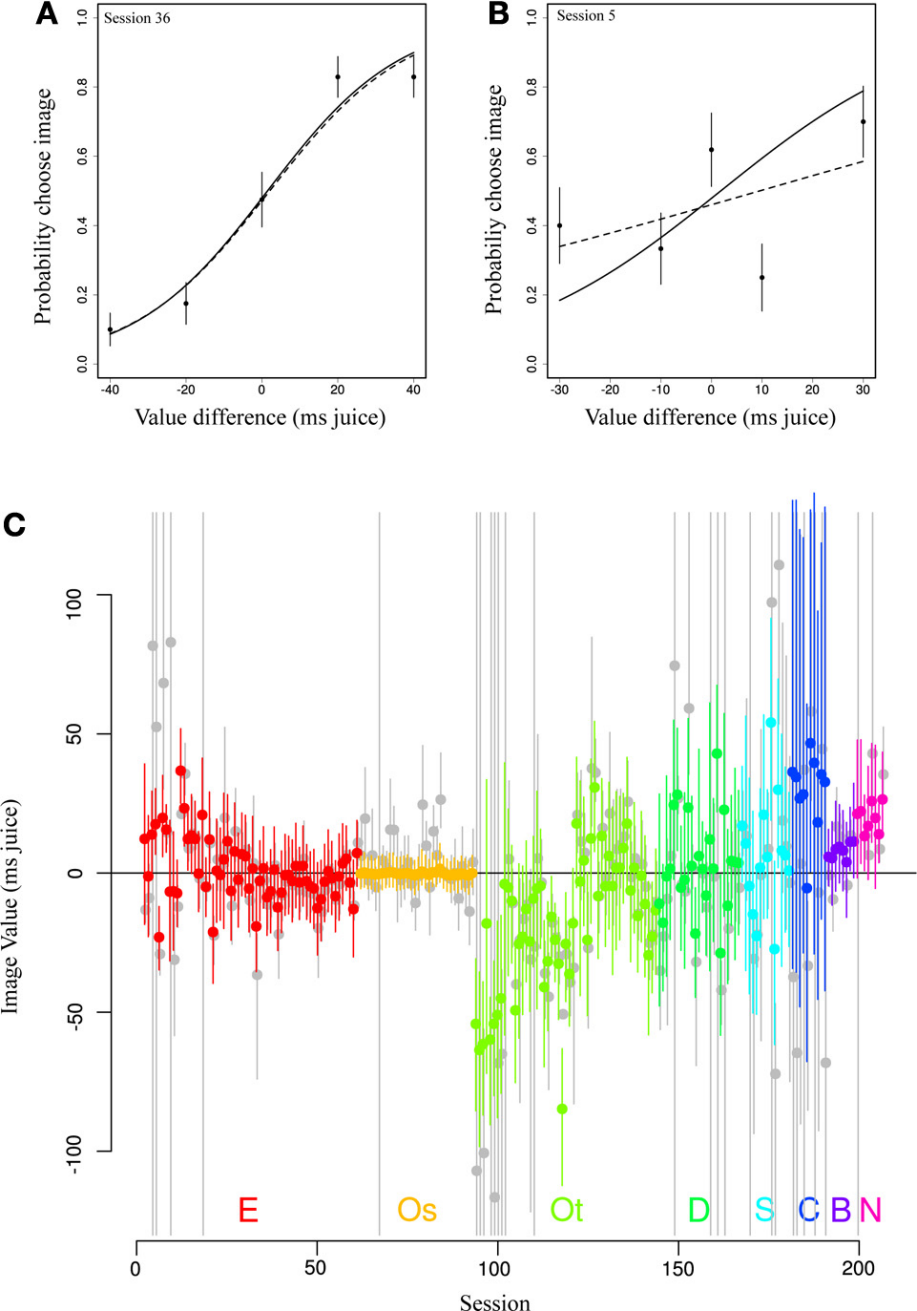
**Partial pooling across sessions regularizes daily estimates of image value. (A,B)** Data (points) depict observed choice behavior from a single monkey (E) for a single image category (Female) for two behavioral sessions. Dotted lines represent fits to only that session's data. Solid lines show estimates from the most likely parameter values from the hierarchical model. In the first panel, the estimates are nearly identical. In the second, ill-behaved data are regularized using information from all other sessions in the same animal, resulting in a steeper choice curve. Error bars represent s.e.m. based on a binomial distribution for counts. **(C)** Original fitted values (gray) and partially pooled values (color) for all sessions for a single category (female).

We then asked how the pooling effect of our model altered daily estimates of image value from those using only within-session data (no pooling). Figures 4A,B depicts two example sessions from a single subject (E) and image category (Female). Standard logit choice curve fits to the single session data are indicated by dotted lines, partial pooling estimates from our model by solid lines. In Figure 4A, the data roughly follow a logit choice curve, captured by the dotted line. When prior information based on other sessions' data is included, this line barely changes. By contrast, in Figure 4B, we see the opposite case, in which an unruly fit based on much less data is tamed by the use of large amounts of prior information. Note that, while the dotted line of the conventional model minimizes deviation from the data, the solid line, which takes into account information shared across sessions, adjusts the curve (in particular, its width) in the direction of a more typical session. This latter type of shift is most apparent in Figure 4C, in which we show value estimates for a single category (female perinea) for each session using only single-session data (in gray) and from the hierarchical model (in color). The figure clearly shows that in cases of numerous well-behaved data, estimates are altered little, while in cases of extremely noisy data, unreasonable single-session fits are constrained by the pooling effect to reasonable values.

**Figure 5:**
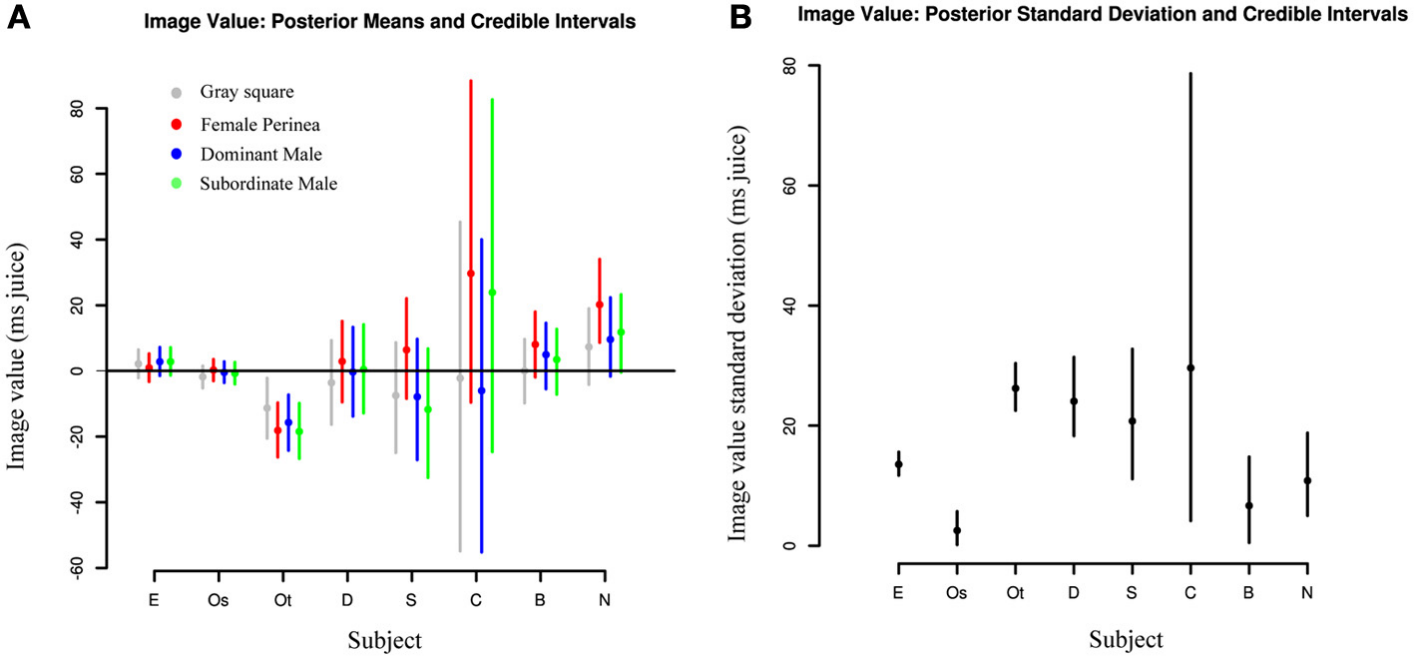
**Image values vary strongly between animals and weakly between image categories. (A)** Comparisons of posterior means for each image category across individuals. Each dot represents the median value across days for a given category of social image for each subject. Lines represent 95% credible intervals. **(B)** Comparisons of posterior standard deviations across individuals. Each dot represents the median standard deviation across days for a given category of social image for each subject. Lines represent 95% credible intervals.

In addition to examining session-to-session variation, we also investigated differences in value distributions between individuals. Figure 5A shows posterior estimates of image value for each category for each subject, along with 95% credible intervals indicating the relative certainty of the estimates. As expected, subjects with fewer sessions had larger credible intervals and thus less reliable estimates. Two observations stand out: first, subjects exhibit marked heterogeneity in social value. While subjects E and Os assigned values near 0 to nearly all categories, subject Ot displayed *negative* values for all categories, indicating that he required *higher* juice to choose the target with the image. Second, subjects are largely consistent in the values they assign across categories. While values may differ markedly across sessions, the overall value distributions for each category substantially overlap, with a trend toward higher value for female images. This further emphasizes the need for accurate daily value estimates, since estimates based on complete pooling may obscure or understate session-to-session differences. Likewise, Figure 5B indicates that there is considerable difference in across-session variation between subjects. That is, while Subject Os has both low image value and low variance across sessions, Subject E has low value with high variance across sessions, and Subject Ot, with consistently negative image values, also has a high daily variance. Indeed, accurate estimates of such variance parameters, a suggested Bayesian analog of ANOVA (Gelman and Hill, 2007), are a key feature of hierarchical models such as ours.

**Figure 6:**
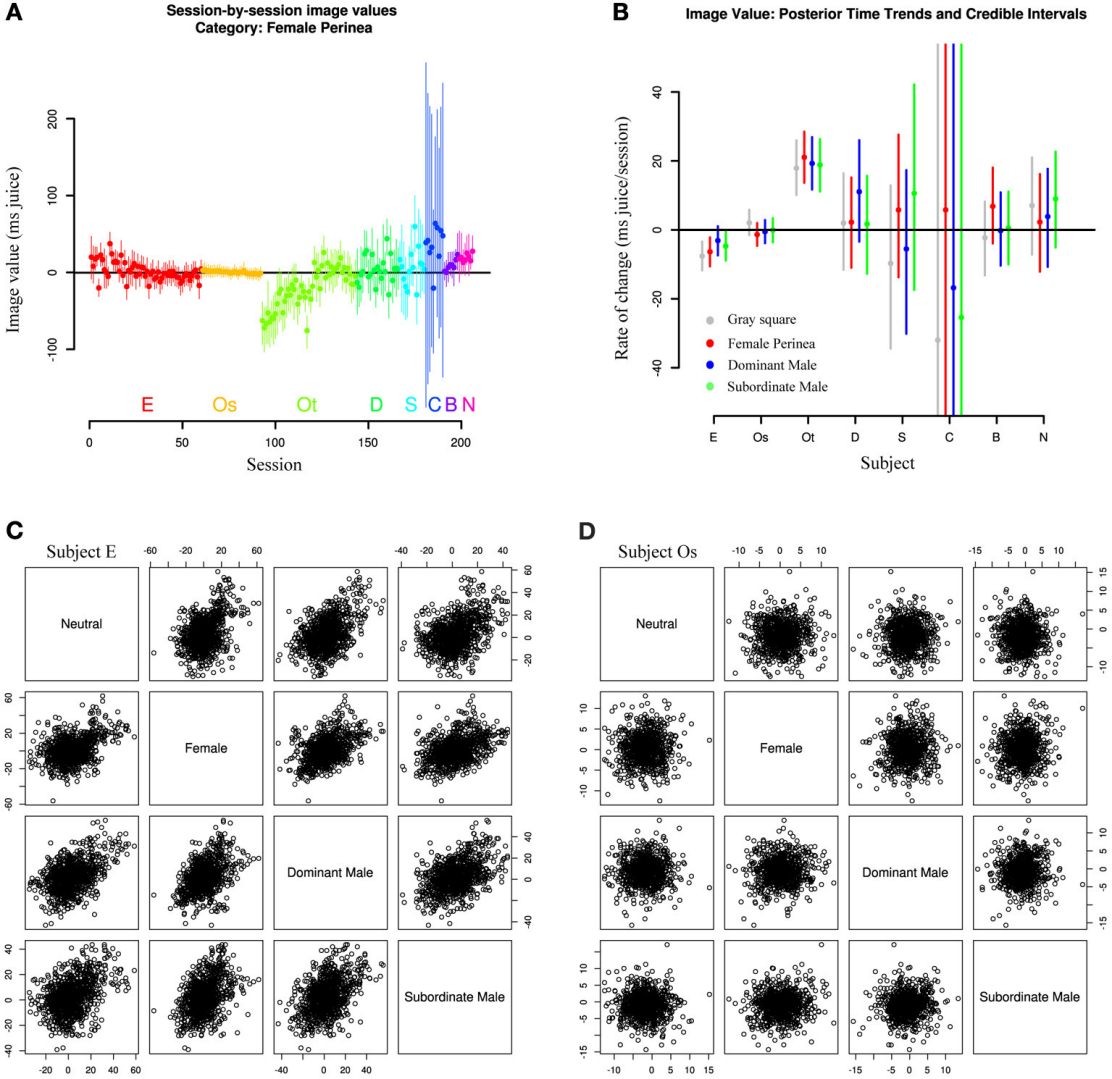
**Image values change with exposure. (A)** Image values for all sessions and subjects for a single image category (female perinea), fit from a model including a linear time trend. Subjects E and Ot show clear time trends across all categories, while other subjects show only limited evidence. **(B)** Posterior medians and credible intervals for time trends across all categories. Lines represent 95% credible intervals. **(C,D)** Scatterplots of image category values across sessions for single subjects E and Os. Individual points are samples drawn from all session posteriors for the given animal. Session-to-session image values are correlated in animals exhibiting time trends in mean image value, but not in animals without time trend. Plots below the diagonal depict the same data as plots above the diagonal with axes flipped.

Finally, we asked whether repeated exposure to our social stimuli resulted in gradual devaluation. This question arose from the observation that Subjects E and Os, with the longest durations of exposure, also showed the smallest absolute mean image values (though not the smallest variation; Figure 5B). To test this hypothesis, we explicitly included session date in our hierarchical model, allowing the mean values for image categories to change over time. As expected, Subject E shows a clear downward trend in image value across all categories over the course of the experiment, with a more muted downward trend in Subject Os (Figure 6A). However, Subject Ot exhibits increased choice for the image option over the course of many sessions, while Subjects D, Sh, C, B, and N have too few sessions to accurately estimate trends. This leaves open two possibilities: One possibility is that subjects may display general desensitizing behavior over time. Alternatively, subjects' changes in image valuation may reflect a changing marginal value of information gain with repeated exposure to the same stimuli.

Moreover, Figure 6B shows that, among subjects with well-estimated change rates (E, Os, Ot), rates of change (slopes in Figure 6A) appear to be correlated within subjects. That is, time trends across sessions seem to be similar across all categories. Subject Ot, for instance, exhibited increasing value not only for female and male images over the course of our experiments, but also for the gray square image, while Subject E exhibits the opposite trend. And while some subjects appear to show selective trends for only single categories (dominant males in Subject D, females in Subject B), these estimates have low confidence, and might not persist in a larger data sample.

A related question is whether values for specific categories are correlated day to day. That is, do we expect a monkey with a strong preference for female images in a given session to likewise exhibit a strong preference for dominant male images? Again, the answer appears to depend on subject. Figure 6C and D show scatterplots of image category value across sessions for two representative subjects (E and Os). Clearly, Subject E exhibits strong correlation among categories, while Subject Os's category values are uncorrelated day-to-day. In fact, these trends may account for previously reported correlations between image values across sessions (Deaner et al., 2005). However, the correlation exhibited by Subject E is likely a byproduct of the overarching time trend, since Subject Os and other monkeys with negligible time trends do not exhibit such correlation.

A potential shortcoming of our results is that they pertain to only a single fitted model (and its extension to time-varying image values) in comparison to only one type of standard regression performed within each session. While Figure 3 appears to show that our model accurately captures features of the data, it remains an open question whether our chosen structure, depicted in Figure 2, includes too many or too few sources of variation. To address these issues, we performed model fits for six additional models, listed in Table 2. Models 0–2 are variants of common regression methods used with similar datasets (Louie and Glimcher, 2010; Pearson et al., 2010). These models either fit a separate choice curve to each session or collapse across sessions to fit a single choice model for each individual. Model 6 is the main model of this paper (Figure 2), and Model 7 includes the time trend. Models 3–5 consider the effects of collapsing the data across individuals, image categories, or both, while still modeling session-to-session variation. These models can be thought of as nested within Model 6, as Model 6 is nested within Model 7. For each model, we calculated the DIC, a generalized goodness-of-fit measure similar to AIC and BIC (Gelman et al., 2003; Berg et al., 2004). Lower numbers indicate better “fits,” meaning that models with low DIC are expected to make better predictions for the values of future data.

**Table 2:** **Model comparisons for distinct combinations of no, partial, and complete pooling of key variables**.

		**Degree of pooling**			**Model measures**		
**Model**	**Description**	**Session**	**Category**	**Subject**	**Deviance ($\bar{D}$)**	***p_D_***	**DIC**
0	All sessions independent	none	none	none	11,906	2401	14,306
1	One model per (category, subject)	complete	none	none	12,017	2392	14,409
2	One model per subject	complete	complete	none	12,022	2413	14,435
3	Only session variation	partial	complete	complete	11,995	2180	14,175
4	Collapse category, model session	partial	complete	none	12,015	2151	14,166
5	Collapse subject, model session	partial	none	complete	11,963	2283	14,246
6	Model session per (category, subject)	partial	none	none	11,974	2221	14,195
7	Model 6 plus time trend	partial	none	none	11,974	2224	14,198

For each model, data for a given variable are either fit separately (no pooling), collapsed (complete pooling) or have their variability modeled (partial pooling). Models 0–2 are the closest to conventional approaches. Model 6 is the model of Figure 2. Model 7 includes a time trend for image value. For model fits, we report the average deviance (−2 times the log likelihood of the data), $\bar{D}$, *p_D_* a measure of the effective number of their parameters, and DIC = $\bar{D}$ + *p_D_*, a measure of model fit. Lower scores indicate models with better generalizability to unobserved data.

From Table 2, it becomes clear that the worst models are those that pool all sessions together, constructing only a single model for each individual or each individual, category pair. This indicates that session-to-session variation is among the most important features of our dataset. Better, but still faring worse than the hierarchical models, is the common strategy of independent fits to each separate data session. Again, this is unsurprising, given that some data sessions contain only three distinct values of *dv*, nearly equal to the number of model parameters. What may be more surprising is that the best-generalizing model is the one in which category information is neglected entirely and session-to-session variation modeled separately for each subject. This reinforces our view that session variation is among the largest sources of observed behavioral variability. It also suggests that models benefit from treating individuals separately, though the second-best fit belongs to the model that collapses across individuals. Finally, our models 6 and 7 perform well, but the DIC analysis suggests they may contain more complexity than is necessary for best prediction. However, it is important to note that only by including this complexity were we able to estimate individual differences for our observed data. Thus, as a matter of practice, it may be necessary to use the larger model for estimation of individual effects and the smaller one for generalizing to new individuals.

## 4. Discussion

By applying Bayesian Hierarchical Modeling to data from a population of rhesus macaques performing multiple sessions of a social valuation choice task, we have demonstrated that hierarchical models can accurately capture the wide range of variation observed both within and between subjects on a daily basis. Moreover, we have shown that by partially pooling data across multiple sessions, we can fit even ill-behaved or extremely limited data, resulting in more robust estimates of model parameters. More importantly, we demonstrated that individual subjects vary widely in the values they assign social images, though across-session variability is just as important as differences between individuals. Our two subjects with the lowest mean value showed low to moderate variance, while our subjects with more extreme values ranged from moderate to high variance. Finally, we showed suggestive evidence in one subject (E) that day-to-day correlations between image values in different categories may be driven by an overall trend toward devaluation of all stimuli, perhaps reflecting the fact that subjects gain less and less unique information from repeated viewing of static images. Viewed this way, the contrary increase in image values exhibited by subject Ot may seem surprising, though this subject, who began the experiment with very few choices of the image option, had a much lower cumulative exposure to images for a given session number than subject E. Indeed, cumulative exposure may be a more accurate predictor of habituation than a simple number of sessions.

In addition, we have shown via model comparison that while our hierarchical model outperforms models that fit each session independently or collapse across all sessions for a single individual, its generalization performance is expected to be poorer than models that ignore subject identity and category and focus on modeling session-to-session variation. However, this result comes with three caveats: First, explicit inclusion of these variables is necessary in cases like ours where the estimation of subject-specific parameters is an analysis goal. Second, as larger and larger numbers of individuals are observed, subject-specific models can more successfully be folded into an extended hierarchical model that explicitly includes variation across the population. Third, these results need not extend to other tasks; social valuation is expected to be variable across individuals to a degree that, say, visual perception is not. The benefit of models such as ours is that this variability, too, can be accurately estimated and compared across both individuals and tasks.

These results reach beyond the immediate social valuation context to model-based inference more generally. As neuroscientific studies increasingly rely on model fits and inferred parameters to characterize behavior, we must accurately account for known sources of variation in choice data. Hierarchical models do so optimally, with the added advantage of providing more informed fits to noisy or ill-behaved data and yielding more accurate subject-level parameters (Gelman et al., 2003; Gelman and Hill, 2007). On a technical note, while these models require a greater investment in time and techniques, multiple software tools exist that substantially lower the level of mathematical sophistication required to implement them (Plummer, 2003; Lunn et al., 2012). Moreover, by requiring us to construct generative models—models explicit about the assumed relations between parameters and data—these methods help refine our thinking and result in models that are easier to interpret. Naturally, the same can be said for more common statistical techniques, but the process of explicit model construction makes us doubly aware of the relationship between our assumptions and the scientific conclusions we draw from them. Most importantly, these models make best use of limited data in studies of individual differences, allowing for more efficient data collection and more robust inference. As a result, they stand to play a key role in the coming, data-rich age of neuroscientific studies.

### Conflict of interest statement

The authors declare that the research was conducted in the absence of any commercial or financial relationships that could be construed as a potential conflict of interest.

## Supplemental data

The combined data, along with code used to produce model fits and figures for the paper, are publicly available: http://www.github.com/jmxpearson/ppv
